# Magnetocaloric Effect in 3D Gd(III)-Oxalate Coordination Framework

**DOI:** 10.3390/nano15010032

**Published:** 2024-12-28

**Authors:** Fang-Wen Lv, Mei-Xin Hong, Xue-Ting Wang, Haiquan Tian, Chun-Chang Wang, Xiu-Ying Zheng

**Affiliations:** 1 Key Laboratory of Structure and Functional Regulation of Hybrid Materials of Ministry of Education, School of Materials Science and Engineering, Institutes of Physical Science and Information Technology, Anhui University, Hefei 230601, China; lfw3214014409@a63.com (F.-W.L.); q21301249@stu.edu.cn (M.-X.H.); 18263446613@163.com (X.-T.W.); 2 Shandong Provincial Key Laboratory of Chemical Energy Storage and Novel Cell Technology, School of Chemistry and Chemical Engineering, Liaocheng University, Liaocheng 252059, China

**Keywords:** magnetocaloric effect, magnetic refrigeration, oxalate, Gd(III)-based complex, 3D framework

## Abstract

Cryogenic magnetic refrigerants based on the magnetocaloric effect (MCE) hold significant potential as substitutes for the expensive and scarce He-3. Gd(III)-based complexes are considered excellent candidates for low-temperature magnetic refrigerants. We have synthesized a series of Ln(III)-based metal-organic framework (MOF) **Ln-3D** (Ln = Gd/Dy) by the slow release of oxalates in situ from organic ligands (disodium edetate dehydrate (EDTA-2Na) and thiodiglycolic acid). Structural analysis shows that the **Ln-3D** is a neutral 3D framework with one-dimensional channels connected by [Ln(H_2_O)_3_]^3+^ as nodes and C_2_O_4_^2−^ as linkers. Magnetic measurements show that **Gd-3D** exhibits very weak antiferromagnetic interactions with a maximum −Δ*S*_m_ value of 36.6 J kg^−1^ K^−1^ (−Δ*S*_v_ = 74.47 mJ cm^−3^ K^−1^) at 2 K and 7 T. The −Δ*S*_m_ value is 28.4 J kg^−1^ K^−1^ at 2 K and 3 T, which is much larger than that of commercial Gd_3_Ga_5_O_12_ (GGG), indicating its potential as a low-temperature magnetic refrigerant.

## 1. Introduction

In molecular magnets, molecule-based materials for magnetic refrigeration via the magnetocaloric effect (MCE) have garnered significant attention due to their benefits, including environmental sustainability and energy efficiency [1]. Magnetic entropy change (−Δ*S*_m_) is a critical metric for assessing MCE performance. A large −Δ*S*_m_ value at low temperatures indicates superior performance of a cryomagnetic refrigerant [2]. High efficiency MCE requires magnetic molecules possessing negative magnetic anisotropy, large total spin ground states and low-lying excited spin states [3]. Among many metals, Gd^3+^ ion, characterized by a high spin state value (S = 7/2) and isotropic nature, makes Gd(III)-based complexes excellent candidates for magnetic refrigeration materials [4,5]. Additionally, weak magnetic interactions between Gd^3+^ ions are conductive to enhancing MCE. Consequently, the cryogenic magnetic refrigeration performance of many Gd(III)-based complexes, including 0D discrete clusters (−Δ*S*_m_ = 41.8 J kg^−1^ K^−1^ of Gd_27_, −Δ*S*_m_ = 43.0 J kg^−1^ K^−1^ of Gd_32_, −Δ*S*_m_ = 39.66 J kg^−1^ K^−1^ of Gd_36_, −Δ*S*_m_ = 38.7 J kg^−1^ K^−1^ of Gd_37_, −Δ*S*_m_ = 43.6 J kg^−1^ K^−1^ of Gd_48_, −Δ*S*_m_ = 48.0 J kg^−1^ K^−1^ of Gd_60_, −Δ*S*_m_ = 46.9 J kg^−1^ K^−1^ of Gd_104_ and −Δ*S*_m_ = 38.0 J kg^−1^ K^−1^ of Gd_140_) [6,7,8,9,10,11,12,13], 1D chains, 2D layers and 3D frameworks (such as −Δ*S*_m_ = 69.5 J kg^−1^ K^−1^ of Gd(CO_3_)F, −Δ*S*_m_ = 76.2 J kg^−1^ K^−1^ of Gd(OH)F_2_ etc.) [14,15,16,17,18,19,20,21,22] have been explored and reported one after another. These studies indicate that increasing magnetic density is one of the key strategies for enhancing −Δ*S*_m_ value.

Magnetic density can be improved by increasing the metal coordination number or reducing the content of non-metal components to raise the metal/ligand ratio. Low-molecular weight ligands with multiple negative charges and high coordination capacity are essential for satisfying the high coordination demands of Gd^3+^ ions and balancing the concentrated positive charges. Currently, Gd(III)-based clusters or Gd(III)-based frameworks constructed with formate, acetate, and carbonate ligands have shown excellent MCE [23,24,25], particularly the large-volume entropy (−Δ*S*_v_) resulting from high metal/ligand ratios. Good candidates for cryogenic magnetic coolers are those complexes in which Gd^3+^ ions’ centers are sufficiently isolated from each other to maintain paramagnetic behavior even at temperatures as low as 2 K. Increasing the dimensionality of Gd-based complexes is an effective approach to enhance magnetic density while ensuring the isolation of Gd^3+^ ions from one another. Unlike these small carboxylate ligands, oxalate is a bridge-like carboxylate ligand featuring rigid coplanar and small steric hindrance. Oxalate offers various coordination modes and can display suitable configurations, forming extended structures by connecting with metal centers, and meeting the high coordination demands of Ln^3+^ ions and controllable structural dimensions of complexes. Investigations showed that the most reported oxalate-based Gd-containing complexes primarily exhibit 1D chain or 2D layer systems [26,27], while only a few cases about 3D metal-organic framework structures have been reported [28,29].

Here, a series of oxalate-based 3D frameworks **Ln-3D** with the formulas {[Ln(C_2_O_4_)_1.5_(H_2_O)_3_]•xH_2_O}_n_ and (Ln = Gd, x = 3, **Gd-3D**; Ln = Dy, x = 5, **Dy-3D**) were obtained using the solvent–thermal reaction of disodium edetate dehydrate (EDTA-2Na), thiodiglycolic acid and Ln(NO_3_)_3_. The C_2_O_4_^2−^ ions were derived from the in situ decomposition of EDTA-2Na and thiodiglycolic acid. Magnetic measurements show that **Gd-3D** exhibits a very weak paramagnetic interaction and **Dy-3D** displays weak antiferromagnetic interaction. **Gd-3D** shows a large −Δ*S*_m_ value of 36.65 J kg^−1^ K^−1^ (−Δ*S*_v_ = 74.47 mJ cm^−3^ K^−1^) at 2 K and 7 T, contributing to the weak paramagnetic interaction and large metal/ligand ratio. Although the −Δ*S*_m_ value of 36.65 J kg^−1^ K^−1^ is slightly slower than that reported for previous Gd(III)-MOF, its −Δ*S*_m_ value of 28.37 J kg^−1^ K^−1^ at 2 K and 3 T is much larger than that of commercial Gd_3_Ga_5_O_12_ (GGG), suggesting its potential as a low-temperature magnetic refrigeration material.

## 2. Materials and Methods

All materials and reagents used were obtained from commercial sources. Magnetic measurements were performed using a Quantum Design MPMSXL magnetometer (San Jose, CA, USA). Powder X-ray diffraction data were collected using a Rigaku Ultima IV powder X-ray diffractometer. The contents of C, H and N were determined using a Vario EL-3 elemental analyzer (EA, El Cajon, CA, USA). The element contents of Gd and Dy were determined using an inductively coupled plasma mass spectrometer (ICP-MS) based on the sample concentration of 20 μg mL^−1^. Fourier transform infrared (FT-IR) spectra were performed on a VERTEX 80 FT−IR spectrophotometer (Boston, MA, USA) with KBr pellets. Thermogravimetric analysis (TGA) was carried out on a thermal analyzer (TGA-5500, TA Instruments, New Castle, DE, USA) under a N_2_ flow rate of 100 L min^−1^ with a heating rate of 3 °C min^−1^.

Synthesis of {[Gd(C_2_O_4_)_1.5_(H_2_O)_3_]•3H_2_O}_n_ (Gd-3D). EDTA-2Na (0.3 mmol, 87.6 mg), thiodiglycolic acid (0.2 mmol, 30.0 mg), Na_2_CO_3_ (0.5 mmol, 52.9 mg), 2-methylimidazole (1.0 mmol, 82.1 mg) and Gd(NO_3_)_3_ (1.0 mL, 1.0 M) were dissolved in deionized water (7.0 mL). The resulting mixture was transferred into a polytetrafluoroethylene liner and then placed in a stainless-steel autoclave resistant to high temperature and high pressure for solvent–thermal reaction. The temperature program used is as follows: the temperature rises to 160 °C over 2 h and is maintained at 160 °C for 72 h; then it gradually cools down to room temperature over 12 h. The resulting reaction solution was filtered and evaporated at room temperature for about 1 week to yield rod-like colorless crystals. Anal. C_3_H_16_GdO_14_: C, 9.06; H, 3.04; Gd, 7.91. Found (%): C, 8.90; H, 3.05; Gd, 8.16. The original data are shown in Figure S5. IR (KBr, cm^−1^): 3356 (s), 1620 (s), 1418 (w), 1384 (m), 1319 (m), 1106 (m), 1053 (w), 1029 (w), 998 (w), 930 (w), 852 (w), 804 (m), 656 (w).

Synthesis of {[Dy(C_2_O_4_)_1.5_(H_2_O)_3_]•5H_2_O}_n_ (Dy-3D). The synthesis steps are the same as for **Gd-3D** with Gd(NO_3_)_3_ replaced by Dy(NO_3_)_3_. Anal. C_3_H_16_DyO_14_: C, 8.21; H, 3.67; Dy, 7.41. Found (%): C, 8.78; H, 2.90; Dy, 7.70. The original data are shown in Figure S5. IR (KBr, cm^−1^): 3356 (s), 1620 (s), 1418 (w), 1384 (m), 1319 (m), 1106 (m), 1053 (w), 1029 (w), 998 (w), 930 (w), 852 (w), 804 (m), 656 (w).

The single crystal data of compounds **Gd-3D** and **Dy-3D** were collected at 120 K using a STOE STADIVARI detector equipped with Cu *K*_α_ radiation (*λ* = 1.54184 Å). Absorption corrections were applied via the multi-scan program STOE LANA (2.4 8.1). The structures were solved by direct methods with hydrogen atoms of the organic ligands generated geometrically (C-H, 0.96 Å). Non-hydrogen atoms were refined anisotropically by least-squares fitting on *F^2^* using the OLEX2 software suite [30,31,32]. Detailed crystallographic data and selected bond lengths and angles are provided in Tables S1–S3. The CCDC numbers of 2401699–2401700 correspond to **Gd-3D** and **Dy-3D**, respectively, and can be accessed free of charge via http://www.ccdc.cam.ac.uk/data_request/cif (accessed on 28 November 2024).

## 3. Results

### 3.1. Synthetic Strategy

The anion-templated method is an effective approach for synthesizing lanthanide clusters. In the process of cluster formation, anions not only act as templates to induce the formation of cluster framework, but also act as counterions to balance the excessive positive charges caused by the aggregation of Ln^3+^ ions, thereby further stabilizing the lanthanide clusters. Particularly, the slow-release anion-templated method, by controlling the rate of anion template formation, facilitates the formation of high-nuclearity metal clusters or high-dimensional cluster-based frameworks. Ligand in situ decomposition is a common method of slow-release anion templating. In this study, although the raw materials EDTA-2Na and thiodiglycolic acid do not directly participate in coordination, the C_2_O_4_^2−^ anions slowly generated through their decomposition under high temperature and pressure act as the sole ligand binding with Ln^3+^ anions. When only H_2_C_2_O_4_ or Na_2_C_2_O_4_ is used in the reaction with Ln^3+^ ions without the presence of EDTA-2Na and thiodiglycolic acid, the target product cannot be obtained. However, when EDTA-2Na, thiodiglycolic acid and Na_2_C_2_O_4_ are present simultaneously, the yield of the target product is significantly improved. Therefore, EDTA-2Na and thiodiglycolic acid also function as buffers during the reaction process.

### 3.2. Crystal Structures

**Figure 1:**
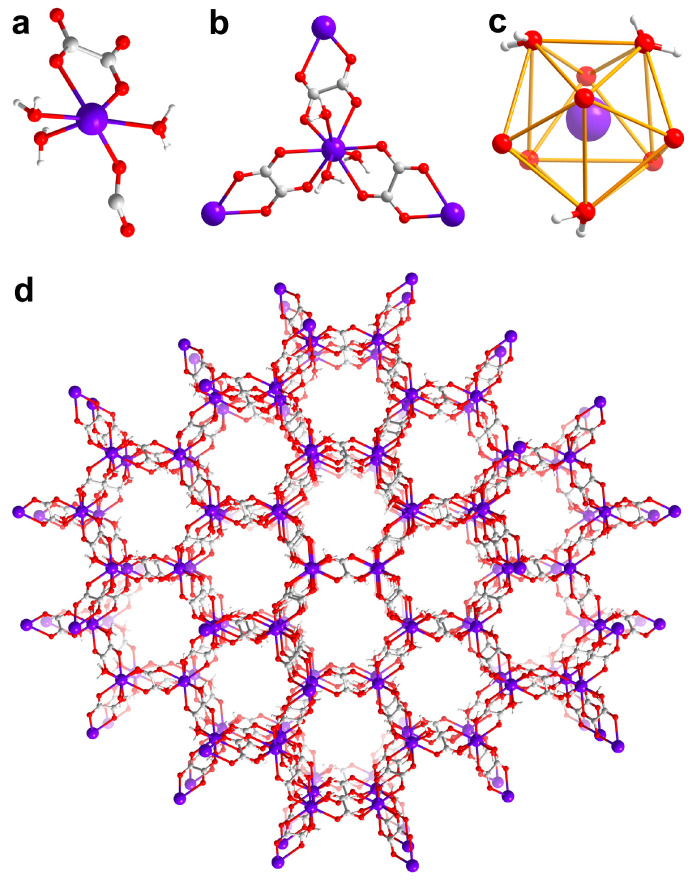
Structural analysis: (**a**) The asymmetric unit [Gd(C_2_O_4_)_1.5_(H_2_O)_3_]. (**b**) The connected mode of C_2_O_4_^2−^ in the 3D framework. (**c**) The coordination mode of Gd^3+^ ion. (**d**) 3D neutral metal framework of **Gd-3D**. Gd, purple. C, gray. O, red. H, white.

The compounds **Gd-3D** and **Dy-3D** are isostructural, both crystallizing in a trigonal crystal system and belonging to the *R*-3 space group. Here, **Gd-3D** is a representative example and its structure will be described in detail. Single-crystal structural analysis shows that **Gd-3D** is a neutral 3D metal framework {[Gd(C_2_O_4_)_1.5_(H_2_O)_3_]•3H_2_O}_n_. As shown in Figure 1a, the asymmetric unit [Gd(C_2_O_4_)_1.5_(H_2_O)_3_] consists of one Gd^3+^ ion, 1.5 C_2_O_4_^2−^ anions and three coordinating water molecules. Single-crystal analysis, combined with EA and TGA data (Figure S1), reveals that each asymmetric unit contains three free water molecules. In **Gd-3D**, C_2_O_4_^2−^ acts both as counter-anions and as linkers, connecting Gd^3+^ ions in the mode of *μ*_4_: *η*^1^: *η*^1^: *η*^1^: *η*^1^ (Figure 1b). Figure 1c shows that Gd^3+^ ion has a nine-coordination with tricapped trigonal prism geometry, surrounded by three O atoms from three coordinating water molecules and six O atoms from three C_2_O_4_^2−^ anions. The bond length of Gd-O ranges from 2.377(11) to 2.536(11) Å, consistent with the previously reported values for oxalate-based lanthanide complexes [26,27]. The compound forms a 3D framework with a 1D channel, constructed from [Gd(H_2_O)_3_]^3+^ as nodes and C_2_O_4_^2−^ as linkers (Figure 1d). The 3D neutral metal framework not only enhances the dimensionality of the compounds but also eliminates free counter-ions, supporting an improved metal/ ligand ratio.

### 3.3. Magnetic Properties

**Figure 2:**
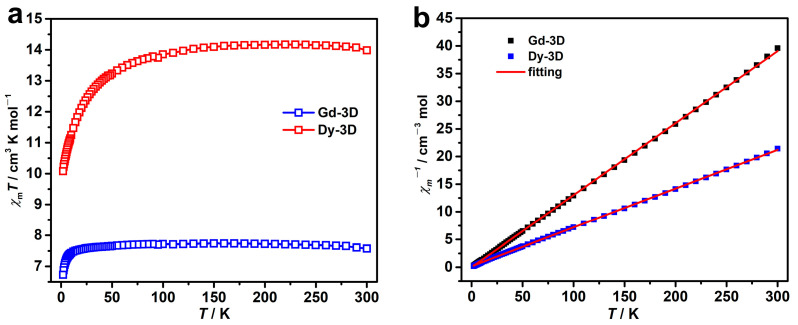
(**a**) Magnetic susceptibilities of **Gd-3D** and **Dy-3D** in the temperature range of 2–300 K with a direct field of 1000 Oe. (**b**) The χ_M_^−1^ *vs T* curves of **Gd-3D** and **Dy-3D** in the range of 2–300 K were fitted using the Curie–Weiss law.

Powder samples of **Gd-3D** and **Dy-3D** were used to measure their magnetic properties. The sample purity was confirmed by powder X-ray diffraction (PXRD). As shown in Figure S2, the experimental PXRD patterns closely match the simulated ones derived from single-crystal data. Magnetic susceptibility measurements of **Gd-3D** and **Dy-3D** were conducted in the temperature range of 2–300 K under a direct current field of 1000 Oe (Figure 2a). At 300 K, the obtained experimental χ_M_*T* values of 7.58 cm^3^ K mol^−1^ for **Gd-3D** and 14.10 cm^3^ K mol^−1^ for **Dy-3D** closely align with the calculated values of 7.87 cm^3^ K mol^−1^ for uncoupled Gd^3+^ (*g* = 2, *J* = 7/2) and 14.16 cm^3^ K mol^−1^ for uncoupled Dy^3+^ (*g* = 4/3, *J* = 15/2), respectively. For **Gd-3D**, the χ_M_*T* value remained almost constant as the temperature decreased to 10 K, and then gradually decreased to 6.9 cm^3^ K mol^−1^ at 2 K, indicating an extremely weak magnetic interaction. For **Dy-3D**, the χ_M_*T* value was almost unchanged from 300 K to 100 K and then slowly decreased to a minimum value of 10.07 cm^3^ K mol^−1^ as the temperature continued to decrease to 2 K, suggesting a weak antiferromagnetic interaction. The χ_M_^−1^ vs. *T* curves of **Gd-3D** and **Dy-3D** in the range of 2–300 K were fitted based on the Curie–Weiss law (Figure 2b), with Curie constants of 7.68 cm^3^ mol^−1^ for **Gd-3D** and 14.27 cm^3^ mol^−1^ for **Dy-3D** and Weiss constants of −0.09 for **Gd-3D** and −2.84 for **Dy-3D**. The near-zero Weiss constant of −0.09 confirms the extremely weak antiferromagnetic interaction in **Gd-3D**, which is conductive to the improvement of MCE. For **Dy-3D**, the negative Weiss constant of −2.84 verifies its antiferromagnetic interaction and zero-field splitting effect.

**Figure 3:**
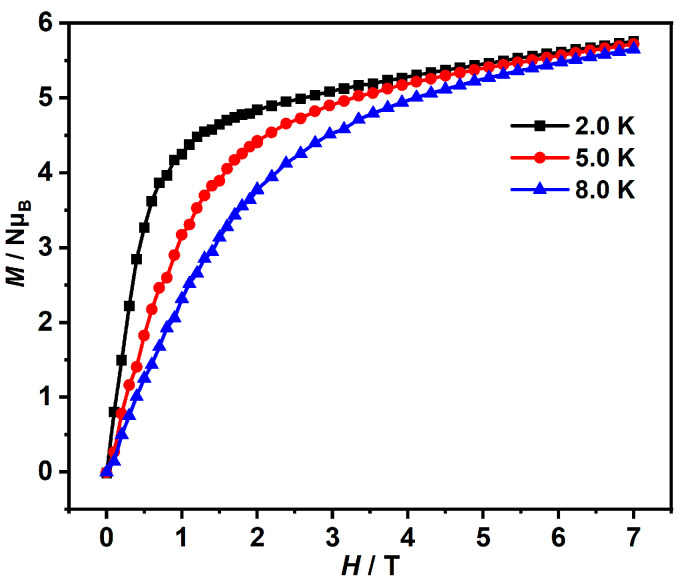
The field-dependent magnetization of **Dy-3D** at 2.0, 5.0 and 8.0 K.

**Figure 4:**
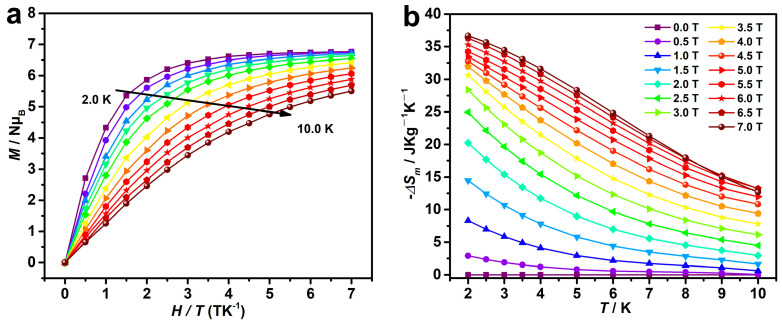
(**a**) The field-dependent magnetization of **Gd-3D** in the range of 2.0–10.0 K. (**b**) −Δ*S*_m_ value of **Gd-3D** at various fields and temperatures.

The field-dependent magnetizations of **Gd-3D** and **Dy-3D** were measured in 0–7 T at different temperatures. The magnetization value of **Dy-3D** was 5.75 *Nμ*_B_ at 2 K and 7 T, which is significantly lower than the theoretical value of 10.00 *Nμ*_B_. Additionally, the magnetization curves of **Dy-3D** at 2 K, 5 K and 7 K do not overlap into a single curve, further confirming the strong magnetic anisotropy and zero-splitting effect in **Dy-3D** (Figure 3). However, the results of alternating current magnetic susceptibilities show that **Dy-3D** shows no frequency-dependent behavior (Figure S3). In contrast, the obtained magnetization value of **Gd-3D** stabilized at approximately 6.76 *Nμ*_B_ at 2 K and 7 T, closely matching the theoretical saturation value of 7.00 *Nμ*_B_, indicating the isotropic nature and extremely weak antiferromagnetic interaction of **Gd-3D** (Figure 4).

The weak magnetic interactions and high metal-to-ligand ratio in **Gd-3D** indicate its potential as an excellent candidate for magnetic refrigeration applications. The −Δ*S*_m_ value is a key parameter in assessing MCE, and it can be estimated from the temperature-dependent magnetizations at 2–7 T using the Maxwell equation ∆*S*_m_(*T*)∆*H* = ∫[∂*M*(*T*, *H*)/∂*T*]*_H_*d*H* [33]. As shown in Figure 4b, the maximum −Δ*S*_m_ value of **Gd-3D** is 36.67 J kg^−1^ K^−1^ at 2 K and 7 T, and the corresponding volume entropy is 74.47 mJ cm^−3^ K^−1^. Compared with previously reported oxalate-based magnetic coolers (Table 1), the −Δ*S*_m_ value of **Gd-3D** is slightly lower than that of the 2D [Gd_2_(C_2_O_4_)_3_(H_2_O)_6_•0.6H_2_O] (−Δ*S*_m_ = 46.6 J kg^−1^ K^−1^) [26], [Gd(C_2_O_4_)(H_2_O)_3_Cl] (−Δ*S*_m_ = 48.0 J kg^−1^ K^−1^) [27], and 3D [Gd(C_2_O_4_)_0.5_(CO_3_)H_2_O] (−Δ*S*_m_ = 50.7 J kg^−1^ K^−1^) [29]. **Gd-3D** exhibits weak antiferromagnetic interactions, while in 3D [Gd(C_2_O_4_)_0.5_(CO_3_)H_2_O] [29], the mixed bridging of CO_3_^2-^ and C_2_O_4_^2-^ between Gd^3+^ ions leads to weak ferromagnetic interactions. For 2D [Gd_2_(C_2_O_4_)_3_(H_2_O)_6_•0.6H_2_O] [26], the planar structure formed by C_2_O_4_^2−^ bridging Gd^3+^ ions ensures good isolation between magnetic centers within the lattice, resulting in weak paramagnetic behavior. These features are beneficial for enhancing the MCE. The −Δ*S*_m_ value of **Gd-3D** is comparable to that of 3D (H_6_edte)_0.5_[Gd(C_2_O_4_)_2_(H_2_O)] [28]. By comparison, both **Gd-3D** and 3D (H_6_edte)_0.5_[Gd(C_2_O_4_)_2_(H_2_O)] contain C_2_O_4_^2−^-bridged Gd^3+^ ions, and 3D (H_6_edte)_0.5_[Gd(C_2_O_4_)_2_(H_2_O)] has a Weiss constant of −0.09 K [28], almost identical to that of **Gd-3D**, showing weak antiferromagnetic interactions in both. Additionally, the crystal density is related to the metal content. The volume entropy (−Δ*S*_V_) is determined by the −Δ*S*_m_ and crystal density. As shown in Table 1, the −Δ*S*_V_ value of **Gd-3D** at 2 K, 7 T is 74.47 mJ cm^−3^ K^−1^, which is lower than that of the reported Gd-based framework materials [25,26,28,29]. This reduction may result from the presence of three free guest water molecules in **Gd-3D**, leading to decreased crystal density and magnetic density.

Relative cooling power (RCP) is another critical parameter used for evaluating magnetocaloric performance, which is calculated as the product of the maximum −Δ*S*_m_ value and the corresponding half-peak width [34]. RCP at low temperatures and low fields is particularly significant for practical applications. At 2 K, the −Δ*S*_m_ value of **Gd-3D** rapidly increases to 20.37 J kg^−1^ K^−1^in the field range from 0.5 to 2.0 T with a corresponding RCP of 68.45 J kg^−1^, comparable to that of the commercial Gd_3_Ga_5_O_12_ (GGG) at 1.2 K and 2 T [1]. The −Δ*S*_m_ value of **Gd-3D** rises to 28.37 J kg^−1^ K^−1^at 2 K and 3 T, exceeding that of GGG in the same field [35,36]. The RCP of **Gd-3D** at 2 K and 3 T is 112 J kg^−1^, which is slightly higher than that of other Gd-based complexes but significantly lower than that of GGG. This discrepancy may be attributed to the higher non-metallic content in **Gd-3D** compared to GGG. Nonetheless, the RCP of 198 J kg^−1^for **Gd-3D** at 2 K and 7 T indicates that it remains a potential cryogenic refrigerant.

**Table 1:** Comparison of parameters of selected Gd-based frameworks and other refrigerant materials in the cryogenic zone.

Compound	H (T)	T (K)	−Δ*S*_m_ (J kg^−1^ K^−1^)	−Δ*S*_v_ (mJ cm^−3^ K^−1^)	RCP (J kg^−1^)	Ref.
**Gd-3D**	1	2	8.32	16.91	23.71	this work
**Gd-3D**	2	2	20.22	41.09	68.45	this work
**Gd-3D**	3	2	28.37	57.65	112.01	this work
**Gd-3D**	4	2	31.96	64.94	146.25	this work
**Gd-3D**	5	2	33.44	67.95	171.17	this work
**Gd-3D**	6	2	35.27	71.67	188.99	this work
**Gd-3D**	7	2	36.67	74.47	198.25	this work
3D (NH_4_)[Gd(C_2_O_4_)(SO_4_)(H_2_O)]	7	2	42.4	137	―	[29]
3D [Gd(C_2_O_4_)_0.5_(CO_3_)(H_2_O)]	7	2	50.7	165	―	[29]
3D (H_6_edte)_0.5_[Gd(C_2_O_4_)_2_(H_2_O)]	9	2	36.8	84.4	―	[28]
2D [Gd_2_(C_2_O_4_)_3_(H_2_O)_6_•0.6H_2_O]	7	2	46.6	139.9	―	[26]
2D [Gd(C_2_O_4_)(H_2_O)_3_Cl]	7	2.2	48.0	144	―	[25]
Gd_3_Ga_5_O_12_ (GGG)	3	1.2	24	173	―	[35,36]
Gd(HCOO)_3_	2	1.1	43.6	168.5	135.47	[23]
[{Gd(OAc)_3_(H_2_O)_2_}_2_]•4H_2_O	2	0.9	32.6	66.5	104.41	[37]
[Gd(HCOO)(OAc)_2_(H_2_O)_2_]	2	0.9	37.0	88.9	118.6	[24]
Gd_2_(fum)_3_(H_2_O)_4_•3H_2_O	2	1.0	18.0	45.3	43.2	[16]
Gd_3_Ga_5_O_12_ (GGG)	2	1.2	20.4	145	67.58	[1]

H_4_edte = N,N,N’,N’’-tetrakis(2-hydroxyethyl)ethlenediamine; fum = fumarate.

## 4. Conclusions

In this study, we have investigated the influence of magnetic interactions and magnetic density on the MCE of **Gd-3D**. The results of magnetic susceptibilities indicate that **Gd-3D** exhibits very weak antiferromagnetic interactions, which is beneficial for enhancing MCE. The −Δ*S*_m_ value of **Gd-3D** is 36.67 J kg^−1^ K^−1^, which is slightly lower than that of the reported oxalate-based Gd(III) complexes, attributed to the presence of additional free water molecules reducing its magnetic density. However, finding low-temperature magnetic refrigerants with an excellent MCE at low fields is even more critical. Compound **Gd-3D** displays a −Δ*S*_m_ value of 28.37 J kg^−1^ K^−1^ at 2 K and 3 T, surpassing that of commercial GGG, indicating that it still meets requirements as a potential low-temperature magnetic refrigerant.

## Data Availability

The data presented in this study are available on request from the corresponding author.

## Supplementary Materials

The following supporting information can be downloaded at: https://www.mdpi.com/article/10.3390/nano15010032/s1, Figure S1: The experimental and simulated PXRD spectra of **Gd-3D** and **Dy-3D**, respectively; Figure S2: The thermogravimetric analysis of (a) **Gd-3D** and (b) **Dy-3D** in N_2_ atmosphere; Figure S3: The alternate current magnetic susceptibilities (a) in-phase and (b) out-of -phase of **Dy-3D** under dc field of 1000 Oe; Figure S4: The IR spectra of **Gd-3D** and **Dy-3D**; Figure S5: (a) The original data of EA for **Gd-3D** (NO. 46) and **Dy-3D** (NO. 47). (b) The original data of ICP-MS for **Gd-3D** (NO. 4) and **Dy-3D** (NO. 3) based on the concentration of 20 μg mL^−1^; Table S1: Crystallographic data for compounds **Ln-3D** (Ln = Gd/Dy); Table S2: Selected bond distances (Å) and band angles (^o^) of **Gd-3D**; Table S3: Selected bond distances (Å) and band angles (^o^) of **Dy-3D**.
