# Supplementary material for: Magnetocaloric Effect in 3D Gd(III)-Oxalate Coordination Framework
Source: Nanomaterials (Basel). 2024 Dec 28;15(1):32. doi: 10.3390/nano15010032 (PMC11722047; doi:10.3390/nano15010032)
Supplement: Supplementary file 1 [file nanomaterials-15-00032-s001.zip › supporting information-R.3 with yellow highlight.pdf]

Electronic Supplementary Information

Magnetocaloric Effect in 3D Gd(III)-Oxalate Coordination Framework

Fang-Wen Lv,[1†] Mei-Xin Hong,[1†] Xue-Ting Wang,[1] Haiquan Tian,[2*] Chun-Chang Wang,[1*] and Xiu-Ying Zheng[1*]

[1] School of Materials Science and Engineering, Institutes of Physical Science and Information Technology, Key Laboratory of Structure and Functional Regulation of Hybrid Materials of Ministry of Education, Anhui University, Hefei, 230601, P. R. China

[2] Shandong Provincial Key Laboratory of Chemical Energy Storage and Novel Cell Technology, School of Chemistry and Chemical Engineering, Liaocheng University, Liaocheng, 252059, P. R. China

Email: tianhaiquan@lcu.edu.cn, ccwang@ahu.edu.cn, xyzheng@ahu.edu.cn

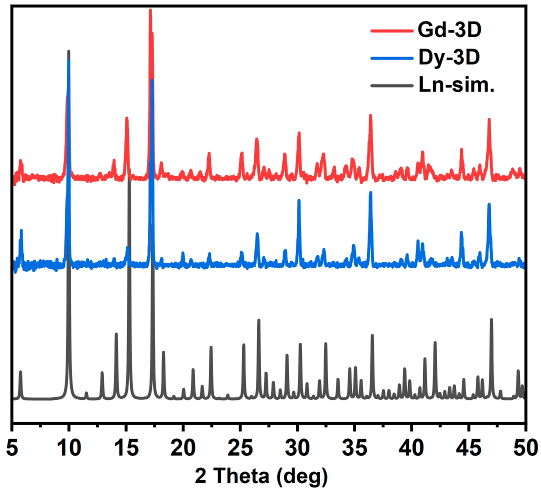

Figure S1 The experimental and simulated PXRD spectra of **Gd-3D** and **Dy-3D**, respectively.

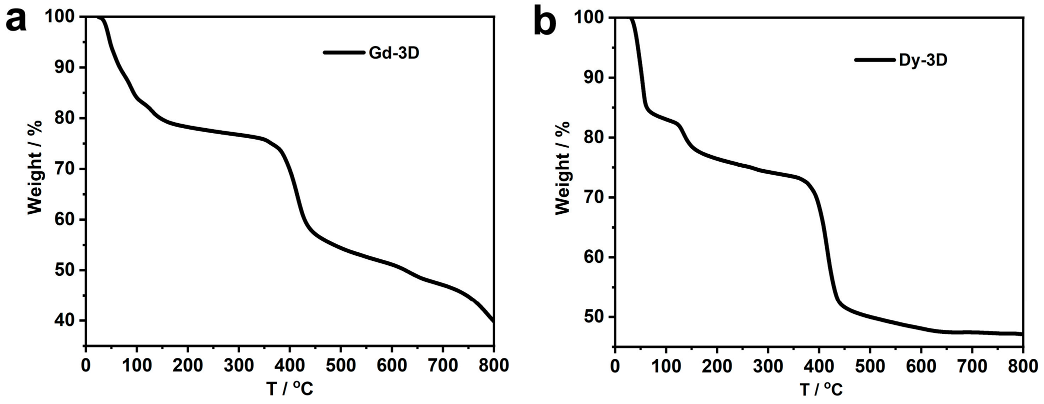

Figure S2 The thermogravimetric analysis of (**a**) **Gd-3D** and (**b**) **Dy-3D** in N_2 atmosphere.

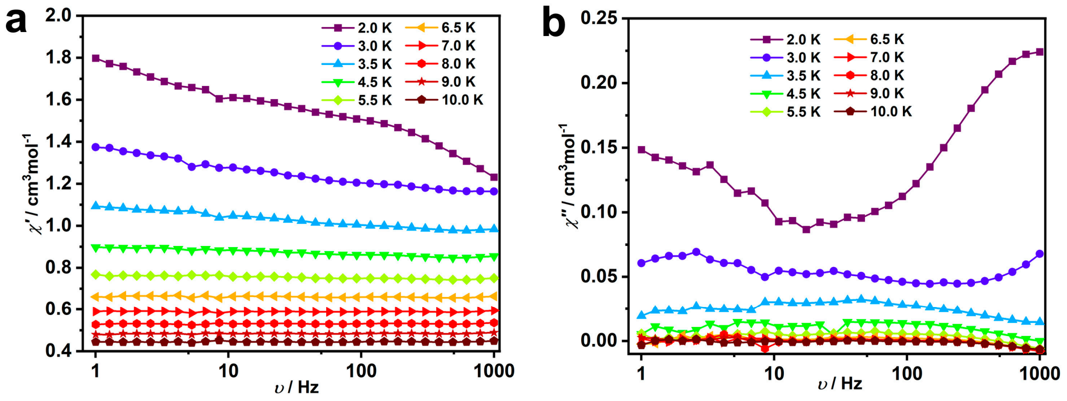

Figure S3 The alternate current magnetic susceptibilities (**a**) in-phase and (**b**) out-of -phase of **Dy-3D** under dc field of 1000 Oe.

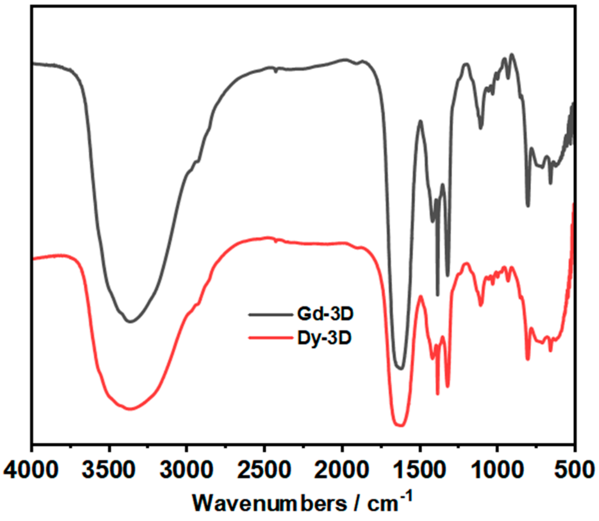

Figure S4 The IR spectra of **Gd-3D** and **Dy-3D**.

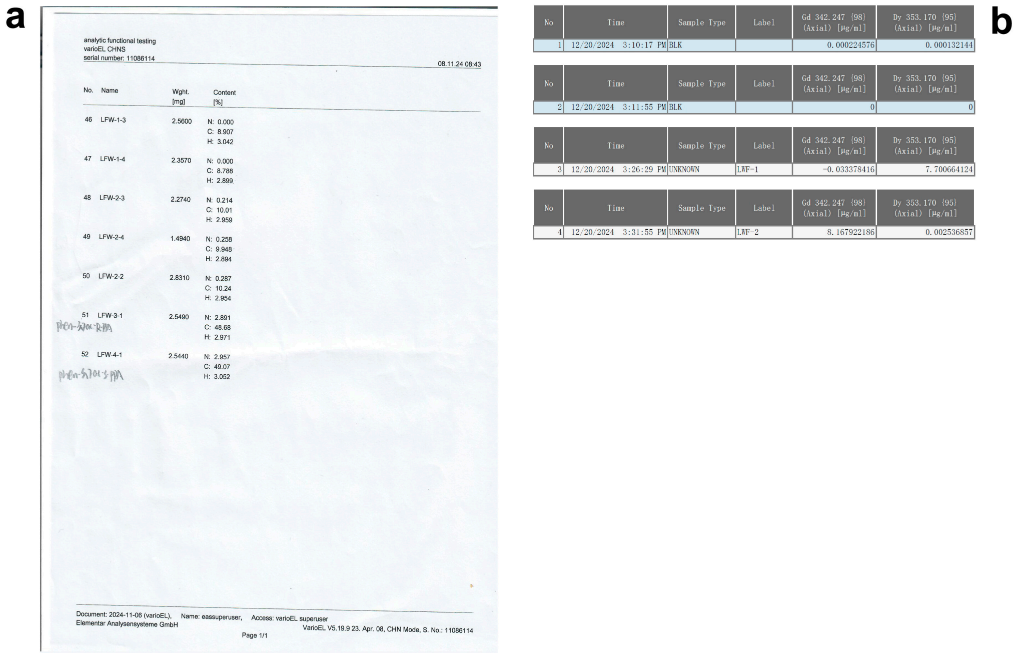

analytic functional testing
varioEL CHNS
serial number: 11086114

08.11.24 08:43

No.	Name	Wght. [mg]	Content [%]
46	LFW-1-3	2.5600	N: 0.000<br>C: 8.907<br>H: 3.042
47	LFW-1-4	2.3570	N: 0.000<br>C: 8.788<br>H: 2.899
48	LFW-2-3	2.2740	N: 0.214<br>C: 10.01<br>H: 2.959
49	LFW-2-4	1.4940	N: 0.258<br>C: 9.948<br>H: 2.894
50	LFW-2-2	2.8310	N: 0.287<br>C: 10.24<br>H: 2.954
51	LFW-3-1	2.5490	N: 2.891<br>C: 48.68<br>H: 2.971
52	LFW-4-1	2.5440	N: 2.957<br>C: 49.07<br>H: 3.052

Document: 2024-11-06 (varioEL), Name: eassuperuser, Access: varioEL superuser
Elementar Analysensysteme GmbH
VarioEL V5.19.9 23. Apr. 08, CHN Mode, S. No.: 11086114
Page 1/1

No	Time	Sample Type	Label	Gd 342.247 {98} (Axial) [µg/ml]	Dy 353.170 {95} (Axial) [µg/ml]
1	12/20/2024 3:10:17 PM	BLK		0.000224576	0.000132144
2	12/20/2024 3:11:55 PM	BLK		0	0
3	12/20/2024 3:26:29 PM	UNKNOWN	LWF-1	-0.033378416	7.700664124
4	12/20/2024 3:31:55 PM	UNKNOWN	LWF-2	8.167922186	0.002536857

Figure S5 (**a**) The original data of EA for **Gd-3D** (NO. 46) and **Dy-3D** (NO. 47). (**b**) The original data of ICP-MS for **Gd-3D** (NO. 4) and **Dy-3D** (NO. 3) based on the concentration of 20 μg mL^{-1}.

Table S1. Crystallographic data for compounds **Ln-3D** (Ln = Gd/Dy).

Compound	**Gd-3D**	**Dy-3D**
Formula	$C_3H_{16}GdO_{12}$	$C_3H_{16}DyO_{14}$
FW	397.38	438.66
T/K	120	120
Cry. system	trigonal	trigonal
Space group	*R*-3	*R*-3
a /Å	30.8062(12)	30.6815(13)
b /Å	30.8062(12)	30.6815(13)
c /Å	7.1104(3)	7.0942(3)
α/°	90	90
β/°	90	90
γ/°	120	120
V/Å^3	5843.9(5)	5783.5(5)
Z	18	18
ρ_c/g cm^{-3}	2.032	2.267
μ/ mm^{-1}	33.551	31.819
Data/parameters	2373/153	3579/182
2θ/°	9.946-139.52	9.986-129.996
Obs. reflections	7087	7889
F(000)	3420	3816
GOF	1.047	1.220
R_1[I> 2σ(I)][a]	0.0768	0.0974
wR_2(All data)[b]	0.2151	0.2905

[a]$R_1=\Sigma||F_O| - |F_C||/\Sigma|F_O|$; [b]$wR_2=\{\Sigma[w(F_O^2 - F_C^2)^2]/\Sigma[w(F_O^2)^2]\}^{1/2}$

Table S2. Selected bond distances (Å) and band angles (°) of **Gd-3D**.

Gd1-O1	2.451(8)	Gd1-O9	2.372(9)
Gd1-O2	2.409(8)	Gd1-O6^{1}	2.511(8)
Gd1-O3	2.432(8)	Gd1-O8^{2}	2.470(8)
Gd1-O4	2.404(8)	Gd1-O11	2.529(9)

Symmetry code:^{1}1-X,1-Y,2-Z; ^{2}1/3+Y,2/3-X+Y,8/3-Z.

O1-Gd1-O6^{1}	64.8(3)	O6^{1}-Gd1-O11	119.2(2)
O1-Gd1-O8^{2}	69.8(3)	O8^{2}-Gd1-O4	138.5(3)
O1-Gd1-O11	143.0(3)	O11-Gd1-O6^{1}	119.2(2)
O2-Gd1-O1	87.4(3)	O7^{2}-Gd1-O1	134.7(3)
O2-Gd1-O3	140.4(3)	O7^{2}-Gd1-O3	138.4(3)
O2-Gd1-O6^{1}	68.6(3)	O7^{2}-Gd1-O6^{1}	137.6(3)
O2-Gd1-O7^{2}	74.9(3)	O7^{2}-Gd1-O8^{2}	65.2(3)
O2-Gd1-O8^{2}	73.2(3)	O7^{2}-Gd1-O11	70.0(3)
O2-Gd1-O11	129.3(3)	O8^{2}-Gd1-O11	120.1(2)
O3-Gd1-O1	79.0(3)	O8^{2}-Gd1-O6^{1}	120.6(2)
O3-Gd1-O6^{1}	72.0(3)	O9-Gd1-O1	120.1(3)
O3-Gd1-O8^{2}	133.1(3)	O9-Gd1-O2	141.6(3)
O3-Gd1-O11	69.1(3)	O9-Gd1-O3	72.4(3)
O4-Gd1-O1	133.2(3)	O9-Gd1-O4	137.9(3)
O4-Gd1-O2	74.3(3)	O9-Gd1-O6^{1}	133.9(3)
O4-Gd1-O3	88.4(3)	O9-Gd1-O7^{2}	88.3(3)
O4-Gd1-O6^{1}	68.5(3)	O9-Gd1-O8^{2}	68.5(3)
O4-Gd1-O7^{2}	81.8(3)	O9-Gd1-O11	72.5(3)

Symmetry code:^{1}1-X,1-Y,2-Z; ^{2}1/3+Y,2/3-X+Y,8/3+Z; ^{3}1/3-Y+X,-1/3+X,8/3-Z

Table S3. Selected bond distances (Å) and band angles (°) of **Dy-3D**.

Dy1-O1	2.409(12)	Dy1-O5^{2}	2.465(11)
Dy1-O2	2.431(11)	Dy1-O6^{2}	2.424(12)
Dy1-O3^{1}	2.505(11)	Dy1-O7	2.345(13)
Dy1-O4	2.542(11)	Dy1-O8	2.371(11)
Symmetry code:^{1}1-X,1-Y,1-Z; ^{2}2/3-Y+X,1/3+X,1/3-Z			
O1-Dy1-O2	79.3(4)	O7-Dy1-O4	73.3(4)
O1-Dy1-O3^{1}	72.8(4)	O7-Dy1-O5^{2}	67.4(4)
O1-Dy1-O4	68.8(4)	O7-Dy1-O6^{2}	88.6(4)
O1-Dy1-O5^{2}	132.5(4)	O7-Dy1-O8	138.7(4)
O1-Dy1-O6^{2}	137.7(4)	O7-Dy1-O12	141.3(4)
O2-Dy1-O3^{1}	65.6(3)	O8-Dy1-O1	88.7(4)
O2-Dy1-O4	142.7(4)	O8-Dy1-O2	134.1(4)
O2-Dy1-O5^{2}	69.0(4)	O8-Dy1-O3^{1}	68.5(4)
O3^{1}-Dy1-O4	119.3(3)	O8-Dy1-O4	65.6(4)
O5^{2}-Dy1-O3^{1}	120.4(3)	O8-Dy1-O5^{2}	138.7(4)
O5^{2}-Dy1-O4	120.3(3)	O8-Dy1-O6^{2}	81.0(4)
O6^{2}-Dy1-O2	135.0(4)	O8-Dy1-O12	73.9(4)
O6^{2}-Dy1-O3^{1}	137.0(4)	O12-Dy1-O1	140.3(4)
O6^{2}-Dy1-O4	69.6(4)	O12-Dy1-O2	87.6(4)
O6^{2}-Dy1-O5^{2}	66.3(4)	O12-Dy1-O3^{1}	67.7(4)
O7-Dy1-O1	72.7(4)	O12-Dy1-O4	129.4(4)
O7-Dy1-O2	79.2(4)	O12-Dy1-O5^{2}	73.9(4)
O7-Dy1-O3^{1}	134.2(4)	O12-Dy1-O6^{2}	75.4(4)

Symmetry code:^{1}1-X,1-Y,1-Z; ^{2}2/3-Y+X,1/3+X,1/3-Z; ^{3}-1/3+Y,1/3-X+Y,1/3-Z
