# Supplementary material for: Magnetocaloric Effect in 3D Gd(III)-Oxalate Coordination Framework
Source: Nanomaterials (Basel). 2024 Dec 28;15(1):32. doi: 10.3390/nano15010032 (PMC11722047; doi:10.3390/nano15010032)

checkCIF/PLATON report

Structure factors have been supplied for datablock(s) Dy-3D, Gd-3D

THIS REPORT IS FOR GUIDANCE ONLY. IF USED AS PART OF A REVIEW PROCEDURE FOR PUBLICATION, IT SHOULD NOT REPLACE THE EXPERTISE OF AN EXPERIENCED CRYSTALLOGRAPHIC REFEREE.

No syntax errors found. CIF dictionary Interpreting this report

Datablock: Gd-3D

Bond precision:	C-C = 0.0185 A	Wavelength=1.54186	
Cell:	a=30.8062(12)	b=30.8062(12)	c=7.1104(3)
	alpha=90	beta=90	gamma=120
Temperature:	120 K		

	Calculated	Reported
Volume	5843.9(5)	5843.9(5)
Space group	R -3	R -3
Hall group	-R 3	-R 3
Moiety formula	C3 H6 Gd O9, 3(H2 O) [+ solvent]	C3 H6 Gd O9, 3(H2 O)
Sum formula	C3 H12 Gd O12 [+ solvent]	C3 H0.06 Gd O12
Mr	397.38	385.34
Dx,g cm-3	2.033	1.971
Z	18	18
Mu (mm-1)	33.551	33.548
F000	3420.0	3205.0
F000'	3279.27	
h,k,lmax	37,37,8	36,37,8
Nref	2469	2373
Tmin,Tmax	0.469,0.715	1.000,1.000
Tmin'	0.249	

Correction method= # Reported T Limits: Tmin=1.000 Tmax=1.000
AbsCorr = MULTI-SCAN

Data completeness= 0.961 Theta(max)= 69.760

R(reflections)= 0.0768(2055) wR2(reflections)= 0.2162(2373)

S = 1.040 Npar= 153

The following ALERTS were generated. Each ALERT has the format
test-name_ALERT_alert-type_alert-level.
Click on the hyperlinks for more details of the test.

Alert level A

```
PLAT417_ALERT_2_A Short Inter D-H..H-D       H2A      ..H10A     .       1.53 Ang.
                                               1-x,1-y,3-z  =     10_668 Check
```

Author Response: This is caused by the fact that the orienatation of the H atoms is not

```
PLAT417_ALERT_2_A Short Inter D-H..H-D       H3A      ..H5B      .       1.64 Ang.
                                                     x,y,z  =      1_555 Check
```

Author Response: This is caused by the fact that the orienatation of the H atoms is not

```
PLAT417_ALERT_2_A Short Inter D-H..H-D       H3B      ..H5B      .       1.69 Ang.
                                                     x,y,z  =      1_555 Check
```

Author Response: This is caused by the fact that the orienatation of the H atoms is not

Alert level B

```
PLAT417_ALERT_2_B Short Inter D-H..H-D       H3B      ..H9B      .       1.97 Ang.
                                       1/3+x-y,-1/3+x,5/3-z  =     18_546 Check
```

Author Response: This is caused by the fact that the orienatation of the H atoms is not

```
PLAT417_ALERT_2_B Short Inter D-H..H-D       H10B     ..H12A     .       2.09 Ang.
                                                     x,y,z  =      1_555 Check
```

Author Response: This is caused by the fact that the orienatation of the H atoms is not

```
PLAT420_ALERT_2_B D-H Bond Without Acceptor  O12      --H12B     .     Please Check
```

Author Response: This is caused by the fact that the orienatation of the H atoms is not

Alert level C

```
PLAT342_ALERT_3_C Low Bond Precision on  C-C Bonds ...............     0.0185 Ang.
PLAT417_ALERT_2_C Short Inter D-H..H-D       H2B      ..H5A      .       2.12 Ang.
                                        1/3+y,2/3-x+y,8/3-z  =     17_557 Check
```

Author Response: This is caused by the fact that the orienatation of the H atoms is not

```
PLAT911_ALERT_3_C Missing FCF Refl Between Thmin & STh/L=    0.600         38 Report
               -5 10  0,  -4 11  0,  -1 14  0, -12 15  0,  -3 15  0,  -8 16  0,
               -5 34  0, -10 35  0,  -7 10  1,  -6 11  1,  -4 13  1,  -3 14  1,
              -35 24  1, -35 27  1, -12 10  2, -11 11  2,  -2 32  2, -25 32  3,
              -28 28  4, -26 30  4, -14 30  4, -21 32  4, -18 32  4, -22 27  5,
              -19 27  5, -21 28  5, -18 28  5, -20 29  5, -17 29  5, -14 29  5,
               10 16  6, -23 22  6, -21 24  6, -17 25  6, -16 26  6, -13 26  6,
              -21 11  7, -15 20  7,
PLAT918_ALERT_3_C Reflection(s) with I(obs) much Smaller I(calc) .          2 Check
```

Alert level G

```
FORMU01_ALERT_1_G  There is a discrepancy between the atom counts in the
            _chemical_formula_sum and _chemical_formula_moiety. This is
            usually due to the moiety formula being in the wrong format.
            Atom count from _chemical_formula_sum:   C3 H.06 Gd1 O12
            Atom count from _chemical_formula_moiety:C3 H12 Gd1 O12
FORMU01_ALERT_2_G  There is a discrepancy between the atom counts in the
            _chemical_formula_sum and the formula from the _atom_site* data.
            Atom count from _chemical_formula_sum:C3 H.06 Gd1 O12
            Atom count from the _atom_site data:  C3 H12 Gd1 O12
CELLZ01_ALERT_1_G Difference between formula and atom_site contents detected.
CELLZ01_ALERT_1_G ALERT: Large difference may be due to a
            symmetry error - see SYMMG tests
           From the CIF: _cell_formula_units_Z   18
           From the CIF: _chemical_formula_sum  C3 H0.06 Gd O12
           TEST: Compare cell contents of formula and atom_site data

           atom    Z*formula  cif sites diff
           C         54.00     54.00    0.00
           H          1.08    216.00 -214.92
           Gd        18.00     18.00    0.00
           O        216.00    216.00    0.00
PLAT004_ALERT_5_G Polymeric Structure Found with Maximum Dimension          3 Info
PLAT007_ALERT_5_G Number of Unrefined Donor-H Atoms .............          12 Report
              H2A   H2B   H3A   H3B   H9A   H9B   H5A   H5B   H10A  H10B  H12A
              H12B
PLAT041_ALERT_1_G Calc. and Reported SumFormula    Strings  Differ     Please Check
              Calc: C3 H12 Gd O12
              Rep.: C3 H0.06 Gd O12
PLAT072_ALERT_2_G SHELXL First  Parameter in WGHT  Unusually Large       0.15 Report
PLAT083_ALERT_2_G SHELXL Second Parameter in WGHT  Unusually Large     119.28 Why ?
PLAT605_ALERT_4_G Largest Solvent Accessible VOID in the Structure        209 A**3
PLAT794_ALERT_5_G Tentative Bond Valency for Gd1       (III)     .       3.25 Info
PLAT802_ALERT_4_G CIF Input Record(s) with more than 80 Characters          2 Info
PLAT869_ALERT_4_G ALERTS Related to the Use of SQUEEZE  Suppressed          ! Info
PLAT870_ALERT_4_G ALERTS Related to Twinning Effects Suppressed ..          ! Info
PLAT883_ALERT_1_G Absent Datum for _atom_sites_solution_primary ..     Please Do !
PLAT910_ALERT_3_G Missing # of FCF Reflection(s) Below Theta(Min).          1 Note
               -1  2  0,
PLAT912_ALERT_4_G Missing # of FCF Reflections Above STh/L=  0.600         56 Note
PLAT931_ALERT_5_G CIFcalcFCF  Twin Law ( 0 0 1)         Est.d BASF       0.37 Check
PLAT931_ALERT_5_G CIFcalcFCF  Twin Law ( 1-1 0)         Est.d BASF       0.34 Check
PLAT941_ALERT_3_G Average HKL Measurement Multiplicity ..........         3.0 Low
PLAT969_ALERT_5_G The 'Henn et al.' R-Factor-gap value ..........       8.429 Note
              Predicted wR2: Based on SigI**2  2.56 or SHELX Weight 20.89
```

```
  3 ALERT level A = Most likely a serious problem - resolve or explain
  3 ALERT level B = A potentially serious problem, consider carefully
  4 ALERT level C = Check. Ensure it is not caused by an omission or oversight
 21 ALERT level G = General information/check it is not something unexpected

  5 ALERT type 1 CIF construction/syntax error, inconsistent or missing data
 10 ALERT type 2 Indicator that the structure model may be wrong or deficient
  5 ALERT type 3 Indicator that the structure quality may be low
  5 ALERT type 4 Improvement, methodology, query or suggestion
  6 ALERT type 5 Informative message, check
```

Datablock: Dy-3D

Bond precision: C-C = 0.0250 A Wavelength=1.54186

Cell:	a=30.6815(13)	b=30.6815(13)	c=7.0942(3)
	alpha=90	beta=90	gamma=120
Temperature:	120 K		

	Calculated	Reported
Volume	5783.5(5)	5783.5(5)
Space group	R -3	R -3
Hall group	-R 3	-R 3
Moiety formula	C3 H6 Dy O9, 5(H2 O) [+ solvent]	C3 H6 Dy O9, 5(H2 O)
Sum formula	C3 H16 Dy O14 [+ solvent]	C3 H16 Dy O14
Mr	438.66	438.66
Dx,g cm-3	2.267	2.267
Z	18	18
Mu (mm-1)	31.819	31.819
F000	3816.0	3816.0
F000’	3656.83	
h,k,lmax	36,36,8	35,36,8
Nref	2191	3579
Tmin,Tmax	0.489,0.727	0.003,0.138
Tmin’	0.267	

Correction method= # Reported T Limits: Tmin=0.003 Tmax=0.138
AbsCorr = MULTI-SCAN

Data completeness= 1.634 Theta(max)= 64.998

R(reflections)= 0.0974(3041) wR2(reflections)= 0.2905(3579)

S = 1.220 Npar= 182

The following ALERTS were generated. Each ALERT has the format
test-name_ALERT_alert-type_alert-level.
Click on the hyperlinks for more details of the test.

Alert level A

```
PLAT417_ALERT_2_A Short Inter D-H..H-D       H1B      ..H9B       .       1.54 Ang.
                                       -1/3+y,1/3-x+y,1/3-z  =     14_455 Check
```

Author Response: This is caused by the fact that the orienatation of the H atoms is not

```
PLAT417_ALERT_2_A Short Inter D-H..H-D       H7B      ..H11B      .       1.60 Ang.
                                        2/3+x-y,1/3+x,4/3-z  =     15_556 Check
```

Author Response: This is caused by the fact that the orienatation of the H atoms is not

```
PLAT417_ALERT_2_A Short Inter D-H..H-D       H9A      ..H12A      .       1.73 Ang.
                                                      x,y,z  =      1_555 Check
```

Author Response: This is caused by the fact that the orienatation of the H atoms is not

```
PLAT417_ALERT_2_A Short Inter D-H..H-D       H10B     ..H14B      .       1.21 Ang.
                                                    x,y,1+z  =      1_556 Check
```

Author Response: This is caused by the fact that the orienatation of the H atoms is not

Alert level B

```
PLAT342_ALERT_3_B Low Bond Precision on  C-C Bonds ..............      0.025 Ang.
```

Author Response: Moderate data quality in context with disorder.

```
PLAT417_ALERT_2_B Short Inter D-H..H-D       H10A     ..H12B      .       2.07 Ang.
                                                    x,y,1+z  =      1_556 Check
```

Author Response: This is caused by the fact that the orienatation of the H atoms is not

```
PLAT417_ALERT_2_B Short Inter D-H..H-D       H10A     ..H12B      .       2.08 Ang.
                                                1-x,1-y,1-z  =     10_666 Check
```

Author Response: This is caused by the fact that the orienatation of the H atoms is not

Alert level C

```
THETM01_ALERT_3_C  The value of sine(theta_max)/wavelength is less than 0.590
            Calculated sin(theta_max)/wavelength =    0.5878
PLAT084_ALERT_3_C High wR2 Value (i.e. > 0.25) ..................       0.29 Report
PLAT911_ALERT_3_C Missing FCF Refl Between Thmin & STh/L=    0.588         21 Report
                0  1  2,   0  0  3,  -2  4  3,  -4  5  3,  -1  5  3,  -9  6  3,
               -6  6  3,  -3  6  3,  -5  7  3,  -2  7  3,  -2 10  3,  -5 13  3,
               -1  1  4,   0  2  4,   3  5  4,  -6  8  4, -15 28  5, -17 13  6,
              -23 19  6, -18 24  6, -15 24  6,
PLAT918_ALERT_3_C Reflection(s) with I(obs) much Smaller I(calc) .          7 Check
```

Alert level G

```
PLAT004_ALERT_5_G Polymeric Structure Found with Maximum Dimension          3 Info
PLAT007_ALERT_5_G Number of Unrefined Donor-H Atoms .............          16 Report
              H1A   H1B   H7A   H7B   H12A  H12B  H9A   H9B   H10A  H10B  H11A
              H11B  H13A  H13B  H14A  H14B
PLAT072_ALERT_2_G SHELXL First  Parameter in WGHT  Unusually Large       0.20 Report
PLAT605_ALERT_4_G Largest Solvent Accessible VOID in the Structure        132 A**3
PLAT794_ALERT_5_G Tentative Bond Valency for Dy1       (III)     .       2.84 Info
PLAT802_ALERT_4_G CIF Input Record(s) with more than 80 Characters          1 Info
PLAT869_ALERT_4_G ALERTS Related to the Use of SQUEEZE  Suppressed          ! Info
PLAT870_ALERT_4_G ALERTS Related to Twinning Effects Suppressed ..          ! Info
PLAT909_ALERT_3_G Percentage of I>2sig(I) Data at Theta(Max) Still        77% Note
PLAT910_ALERT_3_G Missing # of FCF Reflection(s) Below Theta(Min).          1 Note
               -1  2  0,
PLAT931_ALERT_5_G CIFcalcFCF  Twin Law         [ 0 0 1] Est.d BASF       0.53 Check
PLAT931_ALERT_5_G CIFcalcFCF  Twin Law ( 0 0 1)         Est.d BASF       0.53 Check
PLAT931_ALERT_5_G CIFcalcFCF  Twin Law         [ 0 0 1] Est.d BASF       0.53 Check
PLAT931_ALERT_5_G CIFcalcFCF  Twin Law ( 1 0 0)         Est.d BASF       0.49 Check
PLAT941_ALERT_3_G Average HKL Measurement Multiplicity ..........         2.2 Low
PLAT967_ALERT_5_G Note: Two-Theta Cutoff Value in Embedded .res ..      130.0 Degree
PLAT969_ALERT_5_G The 'Henn et al.' R-Factor-gap value ..........      7.969 Note
              Predicted wR2: Based on SigI**2  3.65 or SHELX Weight 23.85
```

   4 **ALERT level A** = Most likely a serious problem - resolve or explain
   3 **ALERT level B** = A potentially serious problem, consider carefully
   4 **ALERT level C** = Check. Ensure it is not caused by an omission or oversight
  17 **ALERT level G** = General information/check it is not something unexpected

   0 ALERT type 1 CIF construction/syntax error, inconsistent or missing data
   7 ALERT type 2 Indicator that the structure model may be wrong or deficient
   8 ALERT type 3 Indicator that the structure quality may be low
   4 ALERT type 4 Improvement, methodology, query or suggestion
   9 ALERT type 5 Informative message, check

It is advisable to attempt to resolve as many as possible of the alerts in all categories. Often the minor alerts point to easily fixed oversights, errors and omissions in your CIF or refinement strategy, so attention to these fine details can be worthwhile. In order to resolve some of the more serious problems it may be necessary to carry out additional measurements or structure refinements. However, the purpose of your study may justify the reported deviations and the more serious of these should normally be commented upon in the discussion or experimental section of a paper or in the "special_details" fields of the CIF. checkCIF was carefully designed to identify outliers and unusual parameters, but every test has its limitations and alerts that are not important in a particular case may appear. Conversely, the absence of alerts does not guarantee there are no aspects of the results needing attention. It is up to the individual to critically assess their own results and, if necessary, seek expert advice.

Publication of your CIF in IUCr journals

A basic structural check has been run on your CIF. These basic checks will be run on all CIFs submitted for publication in IUCr journals (*Acta Crystallographica*, *Journal of Applied Crystallography*, *Journal of Synchrotron Radiation*); however, if you intend to submit to *Acta Crystallographica Section C* or *E* or *IUCrData*, you should make sure that full publication checks are run on the final version of your CIF prior to submission.

Publication of your CIF in other journals

Please refer to the *Notes for Authors* of the relevant journal for any special instructions relating to CIF submission.

PLATON version of 19/12/2024; check.def file version of 19/12/2024

Datablock Gd-3D - ellipsoid plot

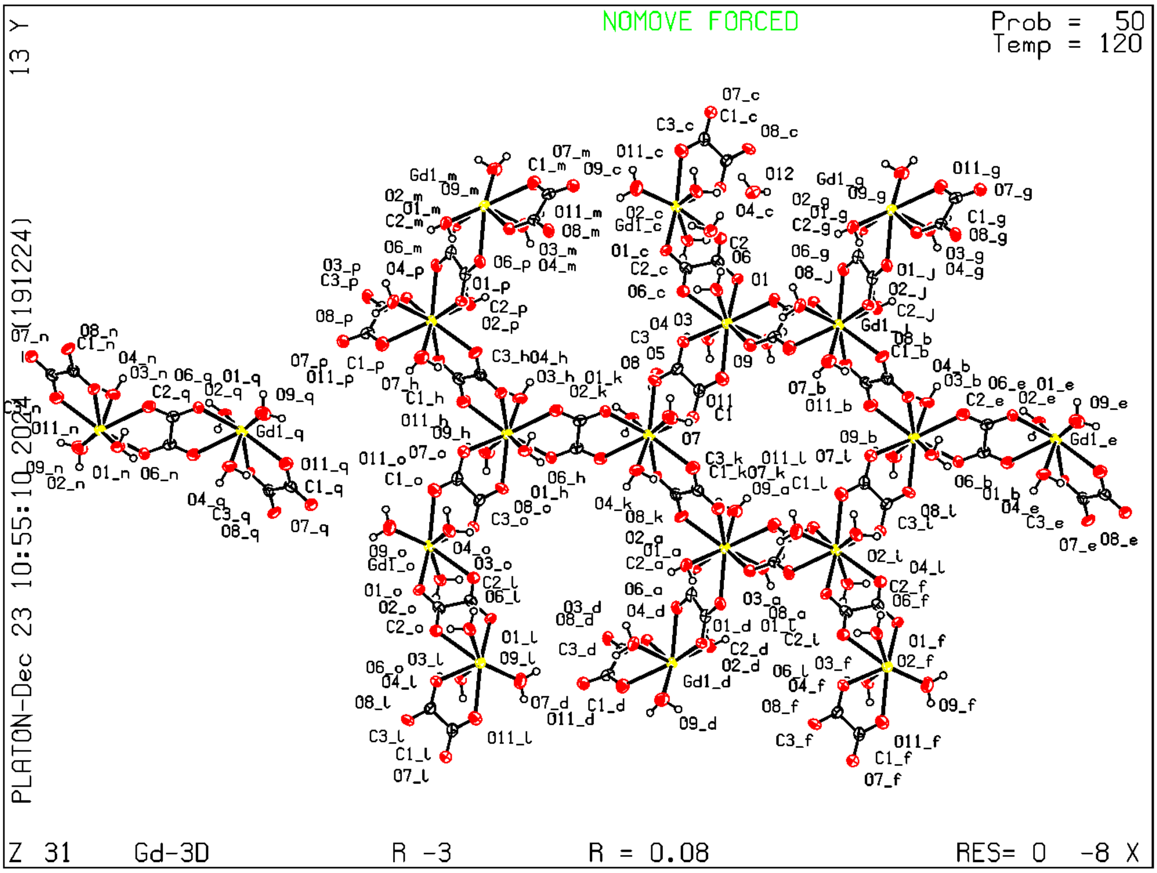

Datablock Dy-3D - ellipsoid plot

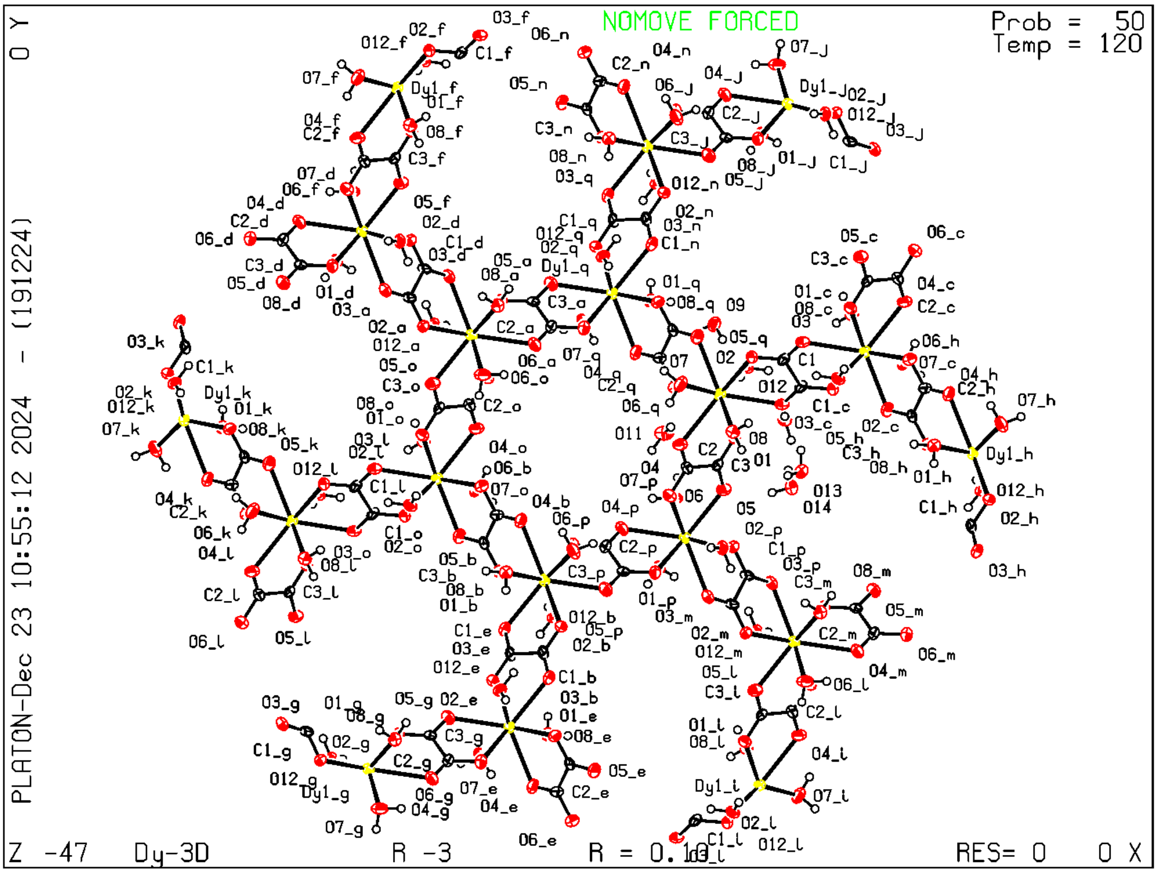

Supplement: Supplementary file 1 [file checkcif.pdf › checkcif.pdf]
